# Supplementary material for: Defining a Characteristic Gene Expression Set Responsible for Cancer Stem Cell-Like Features in a Sub-Population of Ewing Sarcoma Cells CADO-ES1
Source: Int J Mol Sci. 2018 Dec 6;19(12):3908. doi: 10.3390/ijms19123908 (PMC6321634; doi:10.3390/ijms19123908)
Supplement: Supplementary file 1 [file ijms-19-03908-s001.pdf]

International Journal of
Molecular Sciences

MDPI

Article

Defining a Characteristic Gene Expression Set Responsible for Cancer Stem Cell-Like Features in a Sub-Population of Ewing Sarcoma Cells CADO-ES1

Marc Hotfilder, Nikhil Mallela, Jochen Seggewiß, Uta Dirksen and Eberhard Korsching*

Supplementary Figures and Tables

1. *Supplementary Figures*

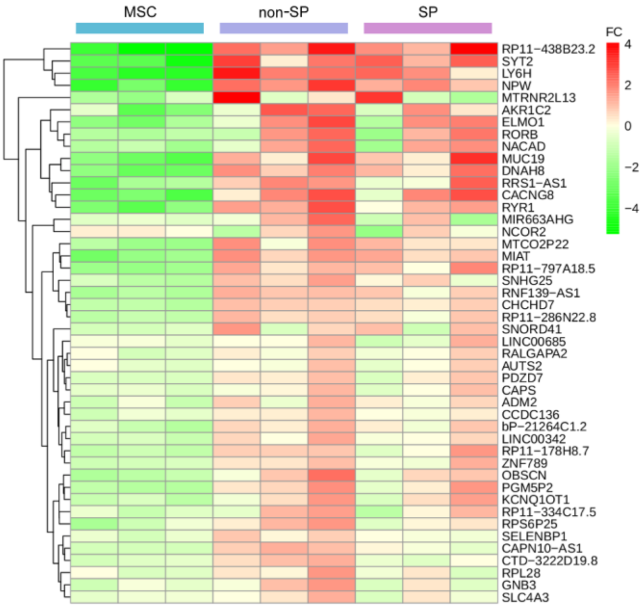

Figure S1. Heatmap of the counts for 46 down-regulated genes from SET-3.
The counts are quantile normalized (preprocessCore) and rlog transformed (DESeq2) prior to plotting. The color bar on the right is defining the fold change values (FC) concerning gene average. Green colors denote levels of down-regulation, while red colors indicate levels of up-regulation. The order of SP and non-SP experiments reflects their paired nature (1,2,3,1,2,3). The hierarchical cluster tree on the left is based on the Euclidean measure.

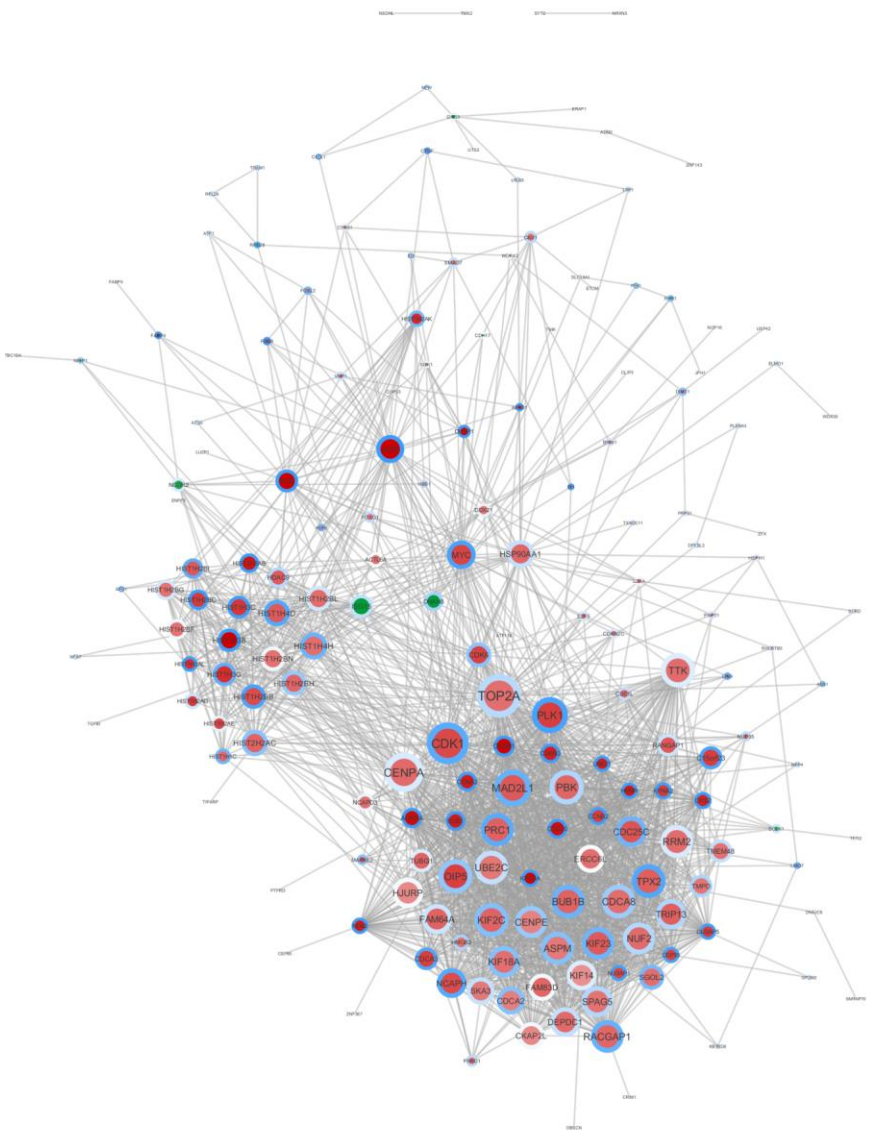

Figure S2. Relevance network based on differential candidates of SET-2 and PPI information of STRINGdb.

The gene products are represented by circles and their interactions are represented by edges. The size of the circles indicate the degree of connectivity to other partners. Larger the circle, greater the degree. Red circles represent the products of up-regulated DEGs and green circles represents products of down-regulated DEGs. The intensity of the colors correspond to the log2 fold changes. Higher the fold change, higher the color intensity. The blue color around the circles represents the P-value. Lower the P-value, higher the color intensity.

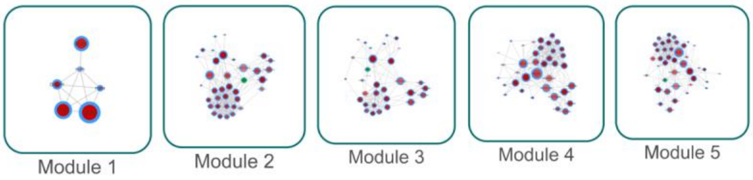

Figure S3. Top 5 highest scoring sub-networks out of the full network.

Module name	jActiveModules score value
Module 1	5.25
Module 2	4.94
Module 3	4.70
Module 4	4.57
Module 5	4.46

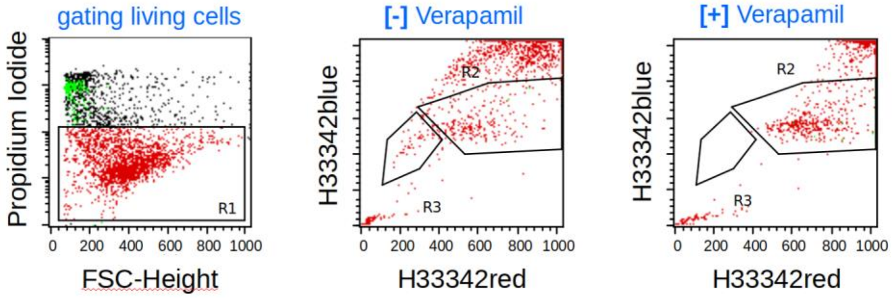

Figure S4. Fluorescence activated cell sorting.
The Hoechst 33342 staining was employed with a concentration of 5µg/ml. The left panel is showing the living cells (gate R1) while the middle and right image shows the difference without Verapamil [-] and with Verapamil (50µM) addition [+]. R2: non-SP gate and R3: SP gate.

Supplementary Tables

Table S1. The top five terms of GO analysis for the up-regulated DEGs using BiNGO application.

Term	Name	Counts	Gene Symbol	p value
Biological Process				
GO:0022403	cell cycle phase	48	SGOL2, CDK6, OIP5, PRC1, ...	2.32e-31
GO:0000279	M phase	44	NCAPD3, CDCA2, CEP55, PRC1, ...	3.10e-31
GO:0006996	organelle organization	75	HIST1H2BN, HIST1H2BF, PRC1, CKS2, ...	3.10e-31
GO:0022402	cell cycle process	52	FAM83D, NCAPH, NUF2, KIF18A, ...	2.31e-30
GO:0000278	mitotic cell cycle	44	CDC20, CDCA2, CCNB1, KIF2C, ...	5.60e-30
Molecular Function				
GO:0005515	protein binding	125	CDK1, LDHA, CDKN2C, CSE1L, ...	1.05e-07
GO:0003677	DNA binding	50	HISTIH2AE, HIST1H3G, NUSAP1, TMPO, ...	5.22e-05
GO:0005488	binding	154	LMO7, HIST1H2BL, HIST1H2AL, TTK, ...	5.22e-05
GO:0003676	nucleic acid binding	58	ZNF480, HISTIH2AK, CENPA, HJURP, ...	1.31e-03
GO:0019899	enzyme binding	19	TOP2A, HIST1H2AG, HDAC9, CDC20, ...	2.84e-03
Cellular Components				
GO:0044427	chromosomal part	44	CENPE, PLK1, HIST1H2BF, KIF2C, ...	4.42e-31
GO:0005694	chromosome	46	HIST1H4D, BUB1, HIST1H3B, CDCA8, ...	1.48e-30
GO:0000786	nucleosome	21	HIST1H3G, HIST2H2AB, HIST1H2BN, HIST1H4H,...	1.28e-23
GO:0005819	spindle	26	DLGAP5, TTK, CDC20, BUB1, ...	2.23e-22
GO:0005634	nucleus	113	HISTIH2AK, EPC1, NOP16, RACGAP1, ...	2.06e-20

Table S2. 43 of 312 DEGs are Oncogenes, fostering tumor stability or progression or suppressing tumor action : a classification breakdown.

	Tumor Promotor Properties	Tumor Suppressor Properties	Both Properties	Total Number
up-regulated	21	8	6	35
down-regulated	1	6	1	8
Total	22	14	7	43

Table S3. The top five GO terms in each GO category having an overlap with study candidates associated with tumor formation or suppression (SET-1 oncogenes).

Term	Name	Gene Symbol	p value
Biological Process			
GO:0022403	cell cycle phase	PLK1 (Both), CDK6 (Both), CCNB2 (Oncogene), CDK1 (Oncogene), CDC25C (Oncogene), PBK (Oncogene), UBE2C (Oncogene), FAM83D (Oncogene), AURKA (Oncogene), CDKN3 (Oncogene), CCNB1 (Oncogene), CDKN2C (Tumor suppressor gene)	2.32e-31
GO:0000279	M phase	PLK1 (Both), CCNB2 (Oncogene), CDK1 (Oncogene), CDC25C (Oncogene), PBK (Oncogene), UBE2C (Oncogene), FAM83D (Oncogene), AURKA (Oncogene), CCNB1 (Oncogene)	3.10e-31
GO:0006996	organelle organization	PLK1 (Both), CAV1 (Both), CXCL1 (Oncogene), CCNB2 (Oncogene), CDK1 (Oncogene), PBK (Oncogene), UBE2C (Oncogene), FAM83D (Oncogene), AURKA (Oncogene), CCNB1 (Oncogene), JUN (Oncogene)	3.10e-31
GO:0022402	cell cycle process	PLK1 (Both), CDK6 (Both), CCNB2 (Oncogene), MYC (Oncogene), CDK1 (Oncogene), CDC25C (Oncogene), PBK (Oncogene), UBE2C (Oncogene), FAM83D (Oncogene), AURKA (Oncogene), CDKN3 (Oncogene), CCNB1 (Oncogene), CDKN2C (Tumor suppressor gene)	2.31e-30
GO:0000278	mitotic cell cycle	PLK1 (Both), CDK6 (Both), CCNB2 (Oncogene), CDK1 (Oncogene), CDC25C (Oncogene), PBK (Oncogene), UBE2C (Oncogene), FAM83D (Oncogene), AURKA (Oncogene), CDKN3 (Oncogene), CCNB1 (Oncogene), CDKN2C (Tumor suppressor gene)	5.60e-30
Molecular Function			
GO:0005515	protein binding	PLK1 (Both), CDK6 (Both), CAV1 (Both), FOXO1 (Both), CTGF (Both), CXCL1 (Oncogene), CCNB2 (Oncogene), ID1 (Oncogene), MYC (Oncogene), CDK1 (Oncogene), CDC25C (Oncogene), ID2 (Oncogene), PBK (Oncogene), UBE2C (Oncogene), ATF1 (Oncogene), E2F5 (Oncogene), KIF14 (Oncogene), COPS3 (Oncogene), SGK1 (Oncogene), AURKA (Oncogene), CDKN3 (Oncogene), CCNB1 (Oncogene), JUN (Oncogene), FOS (Oncogene), SPRY4 (Tumor suppressor gene), LRIG3 (Tumor suppressor gene), CDKN2C (Tumor suppressor gene), DUSP1 |(Tumor suppressor gene)	1.05e-07
GO:0003677	DNA binding	KLF6 (Both), POXO1 (Both), MYC (Oncom (Oncogene), ATF1 (Oncogene), E2F5 (Oncogene), JUN (Oncogene), FOS (Oncogene)	5.22e-05
GO:0005488	binding	PLK1 (Both), KLF6 (Both), CDK6 (Both), CAV1 (Both), FOXO1 (Both), CTGF (Both), CXCL1 (Oncogene), CCNB2	5.22e-05

		(Oncogene), ID1 (Oncogene), MYC (Oncogene), CDK1 (Oncogene), CDC25C (Oncogene), ID2 (Oncogene), PBK (Oncogene), UBE2C (Oncogene), ATF1 (Oncogene), E2F5 (Oncogene), KIF14 (Oncogene), COPS3 (Oncogene), SGK1 (Oncogene), AURKA (Oncogene), CDKN3 (Oncogene), CCNB1 (Oncogene), JUN (Oncogene), FOS (Oncogene), SPRY4 (Tumor suppressor gene), LRIG3 (Tumor suppressor gene), CDKN2C (Tumor suppressor gene), UBE2QL1 (Tumor suppressor gene), DUSP1 |(Tumor suppressor gene)	
GO:0003676	nucleic acid binding	KLF6 (Both), FOXO1 (Both), MYC (Oncogene), ATF1 (Oncogene), E2F5 (Oncogene), JUN (Oncogene), FOS (Oncogene)	1.31e-03
GO:0019899	enzyme binding	PLK1 (Both), CAV1 (Both), AURKA (Oncogene), CCNB1 (Oncogene), CDKN2C (Tumor suppressor gene), DUSP1 |(Tumor suppressor gene)	2.84e-03
Cellular Components			
GO:0044427	chromosomal part	PLK1 (Both), ID2 (Oncogene), CCNB1 (Oncogene), JUN (Oncogene)	4.42e-31
GO:0005694	chromosome	PLK1 (Both), ID2 (Oncogene), CCNB1 (Oncogene), JUN (Oncogene)	1.48e-30
GO:0000786	nucleosome	[no tumor associated overlap]	1.28e-23
GO:0005819	spindle	MYC (Oncogene) CDK1 (Oncogene), KIF14 (Oncogene), FAM83D (Oncogene), AURKA (Oncogene), CCNB1 (Oncogene)	2.23e-22
GO:0005634	nucleus	PLK1 (Both), KLF6 (Both), CDK6 (Both), FOXO1 (Both), CCNB2 (Oncogene), ID1 (Oncogene), MYC (Oncogene), CDK1 (Oncogene), CDC25C (Oncogene), ID2 (Oncogene), UBE2C (Oncogene), ATF1 (Oncogene), E2F5 (Oncogene), KIF14 (Oncogene), COPS3 (Oncogene), SGK1 (Oncogene), AURKA (Oncogene), CCNB1 (Oncogene), JUN (Oncogene), FOS (Oncogene), LIN9 (Tumor suppressor gene), CDKN2C (Tumor suppressor gene), DUSP1 |(Tumor suppressor gene)	2.06e-20

Table S4. The top 10 KEGG and REACTOME pathways having an overlap with study candidates associated with tumor formation or suppression (SET-1 oncogenes).

Identifier	Name	Gene Symbol	p value
KEGG Pathways			
hsa05034	Alcoholism	[no tumor associated overlap]	6.96e-13
hsa05322	Systemic lupus erythematosus	[no tumor associated overlap]	4.37e-13
hsa04110	Cell cycle	PLK1 (Both), CDK6 (Both), CCNB2 (Oncogene), MYC (Oncogene), CDK1 (Oncogene), CDC25C (Oncogene), E2F5 (Oncogene), CCNB1 (Oncogene), CDKN2C (Tumor suppressor gene)	4.30e-09
hsa05203	Viral carcinogenesis	CDK6 (Both), CDK1 (Oncogene), JUN (Oncogene)	3.00e-07
hsa04914	Progesterone-mediated oocyte maturation	PLK1 (Both), CCNB2 (Oncogene), CDK1 (Oncogene), CDC25C (Oncogene), CCNB1 (Oncogene)	1.58e-04
hsa04114	Oocyte meiosis	PLK1 (Both), CDK1 (Oncogene), CDC25C (Oncogene), AURKA (Oncogene)	3.12e-02
hsa05202	Transcriptional misregulation in cancer	FOXO1 (Both), MYC (Oncogene), ID2 (Oncogene), ATF1 (Oncogene), CDKN2C (Tumor suppressor gene)	5.40e-02
hsa04115	p53 signaling pathway	CDK6 (Both), CCNB2 (Oncogene), CDK1 (Oncogene), CCNB1 Oncogene)	1.04e-01
hsa04350	TGF-beta signaling pathway	ID1 (Oncogene), MYC (Oncogene), ID2 (Oncogene), E2F5 (Oncogene)	1.92e-01
hsa05166	HTLV-1 infection	MYC (Oncogene), ATF1 (Oncogene), JUN (Oncogene), FOS (Oncogene), CDKN2C (Tumor suppressor gene)	2.92e-01
REACTOME Pathways			
5334118	DNA methylation	[no tumor associated overlap]	8.55e-15
2299718	Condensation of Prophase Chromosomes	PLK1 (Both), CDK1 (Oncogene), CCNB1 (Oncogene)	8.55e-15
68886	M Phase	PLK1 (Both), CCNB2 (Oncogene), CDK1 (Oncogene), UBE2C (Oncogene), CCNB1 (Oncogene)	8.55e-15
68875	Mitotic Prophase	PLK1 (Both), CCNB2 (Oncogene), CDK1 (Oncogene), CCNB1 (Oncogene)	8.55e-15
2559582	Senecence-	CDK6 (Both), UBE2C (Oncogene), JUN (Oncogene),	8.55e-15

	Associated Secretory Phenotype (SASP)	FOS (Oncogene), CDKN2C (Tumor suppressor gene)	
69278	Cell Cycle, Mitotic	PLK1 (Both), CDK6 (Both), CCNB2 (Oncogene), MYC (Oncogene), CDK1 (Oncogene), CDC25C (Oncogene), UBE2C (Oncogene), E2F5 (Oncogene), AURKA (Oncogene), CCNB1 (Oncogene), LIN9 (Tumor suppressor gene), CDKN2C (Tumor suppressor gene)	8.55e-15
1640170	Cell Cycle	PLK1 (Both), CDK6 (Both), CCNB2 (Oncogene), MYC (Oncogene), CDK1 (Oncogene), CDC25C (Oncogene), UBE2C (Oncogene), E2F5 (Oncogene), AURKA (Oncogene), CCNB1 (Oncogene), LIN9 (Tumor suppressor gene), CDKN2C (Tumor suppressor gene)	8.55e-15
73728	RNA Polymerase I Promoter Opening	[no tumor associated overlap]	8.55e-15
212300	PRC2 methylates histones and DNA	[no tumor associated overlap]	4.13e-14
2559580	Oxidative Stress Induced Senescence	CDK6 (Both), JUN (Oncogene), FOS (Oncogene), CDKN2C (Tumor suppressor gene)	4.13e-14
